# Midterm Outcomes of a Bangladeshi Modification of Bent Ab Interno Needle Goniectomy (B‐BANG): Single‐Surgeon Pilot Study

**DOI:** 10.1155/bmri/4125584

**Published:** 2025-09-21

**Authors:** Md. Iftekher Iqbal, Fariah Osman

**Affiliations:** ^1^ Department of Ophthalmology, Bangladesh Eye Hospital & Institute, Dhaka, Bangladesh; ^2^ Department of Ophthalmology, National Institute of Ophthalmology and Hospital, Dhaka, Bangladesh

**Keywords:** BANG, goniectomy, MIGS, TrabEx+

## Abstract

**Purpose:** The purpose of the study is to report midterm outcomes of a pilot case series using a Bangladeshi modification of bent ab interno needle goniectomy (B‐BANG) in primary open‐angle glaucoma (POAG).

**Methods:** This single‐surgeon, nonrandomized case series included 11 eyes of 11 patients with POAG. Six eyes underwent standalone B‐BANG, while five eyes underwent combined phacoemulsification with B‐BANG. Preoperative and postoperative intraocular pressure (IOP), antiglaucoma medication (AGM) burden, and complications were recorded at 1, 3, and 6 months. Paired *t*‐tests and Cohen’s *d* effect sizes with 95% confidence intervals (CIs) were calculated.

**Results:** Mean IOP reduced from 22.73 ± 7.38 to 17.00 ± 2.19 mmHg at 6 months (*p* = 0.0368; Cohen’s *d* = 0.73). AGM usage decreased from 2.09 ± 0.94 to 0.45 ± 0.52 (*p* < 0.0001; *d* = 2.02). No sight‐threatening complications occurred.

**Conclusions:** B‐BANG was feasible and reduced IOP and medication burden. Findings require confirmation in future controlled studies.

## 1. Introduction

Glaucoma represents a significant contributor to irreversible blindness on a global scale, impacting more than 76 million individuals [1]. Pressure gradient across the trabecular meshwork (TM) due to increased resistance to aqueous humor outflow plays an important role in the pathogenesis of primary open‐angle glaucoma (POAG) and other secondary open‐angle glaucoma [2].

Conventional filtration surgeries, although efficacious, involve potential complications and require considerable postoperative monitoring. On the other hand, minimally invasive glaucoma surgery (MIGS) has developed as a safer alternative to conventional filtration surgeries, specializing in bypassing the TM to improve aqueous outflow [1, 2].

In 1936, the incising TM technique was introduced to establish a continuous pathway between the anterior chamber (AC) and Schlemm’s canal [3]. In recent years, the evolution of the procedure has shifted from addressing childhood glaucomas to focusing on adult glaucoma [4–7]. A variety of ab interno trabeculotomy procedures exist within the scope of MIGS. These procedures encompass incisional goniotomy utilizing devices such as the Trabectome (NeoMedix, Tustin, California, United States), the Kahook Dual Blade (KDB, New World Medical Inc., Rancho Cucamonga, California, United States), and the TrabEx+ (MicroSurgical Technology, Redmond, Washington, United States). Additionally, 360° trabeculotomy, also known as gonioscopy‐assisted transluminal trabeculotomy (GATT), can be performed using either a suture or the iTrack device (iScience Interventional Corp., Menlo Park, California, United States), as well as the TRAB360/OMNI system (Sight Sciences Inc., Menlo Park, California, United States) [8, 9].

The bent ab interno needle goniectomy (BANG) represents an economical MIGS technique that establishes a connection between the AC and Schlemm’s canal [1]. This method involves using a modified hypodermic needle for ab interno goniotomy and has shown potential in lowering intraocular pressure (IOP) and drug usage [9]. However, it requires frequent viscoelastic devices (OVDs) for clearing hyphema to gain access to the area of angle during the procedure.

On the other hand, a dedicated MIGS device for ab interno goniotomy, TrabEx+ (MicroSurgical Technology, Redmond, Washington, United States), has aspiration and irrigation capabilities to keep the operative field free of hyphema for well visualization of the AC angle [2]. Its costly price, however, restricts its use in regions with limited resources.

This pilot study presents a modification of BANG, termed B‐BANG (Bangladeshi modification of bent ab interno needle goniectomy), which introduces continuous irrigation using existing phaco equipment to ensure clear visualization, while blood obscuration during BANG necessitates frequent OVD exchange.

## 2. Materials and Methods

### 2.1. Study Design

This convenience‐based, exploratory pilot study encompassed nonrandomized cases of 11 eyes with mild to moderate POAG without formal sample size calculation that underwent treatment utilizing the B‐BANG technique by a single surgeon (M.I.I.) at Bangladesh Eye Hospital, Uttara Branch, Bangladesh, during the period from July 1, 2023, to March 31, 2024. It adheres to the CONSORT extension for pilot and feasibility trials, aiming to assess procedural feasibility, early clinical outcomes, and guide future research. The research received approval from the Institutional Review Board of our hospital and complied with the principles outlined in the Declaration of Helsinki.

### 2.2. Patients

All patients provided written consent before surgery. Preoperative data included age, gender, best‐corrected visual acuity (BCVA) with Snellen’s chart, IOP with Goldmann applanation tonometry (GAT), antiglaucoma medication (AGM) usage, along with detailed gonioscopy to confirm wide open angle (Shaffer’s Grade 4) and absence of peripheral anterior synechiae (PAS). The retinal nerve fiber layer (RNFL) was examined structurally using optical coherence tomography (OCT) (Spectralis OCT, Heidelberg Engineering, Germany) and functionally utilizing 24‐2 visual field tests with SITA‐Fast (Humphrey Field Analyzer, Carl Zeiss Meditec AG, Germany).

### 2.3. Surgical Technique: B‐BANG

In patients with mild‐to‐moderate POAG, the surgery was performed either alone [3] or in conjunction with cataract surgery [8]. Figure 1 shows the instruments required for B‐BANG.

Figure 1Instruments for B‐BANG. (a, b) 26‐G bent needle. (c) Irrigation tube of phaco machine.

**Figure:**
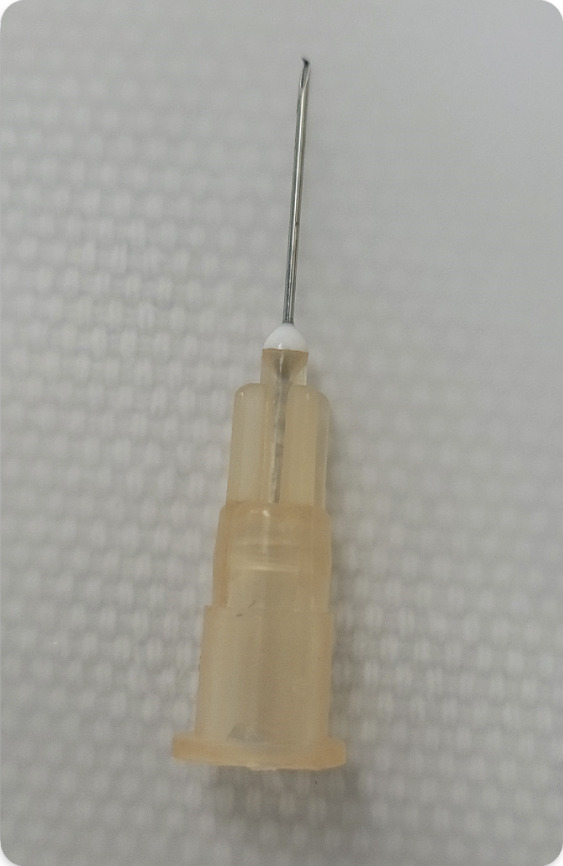
(a)

**Figure:**
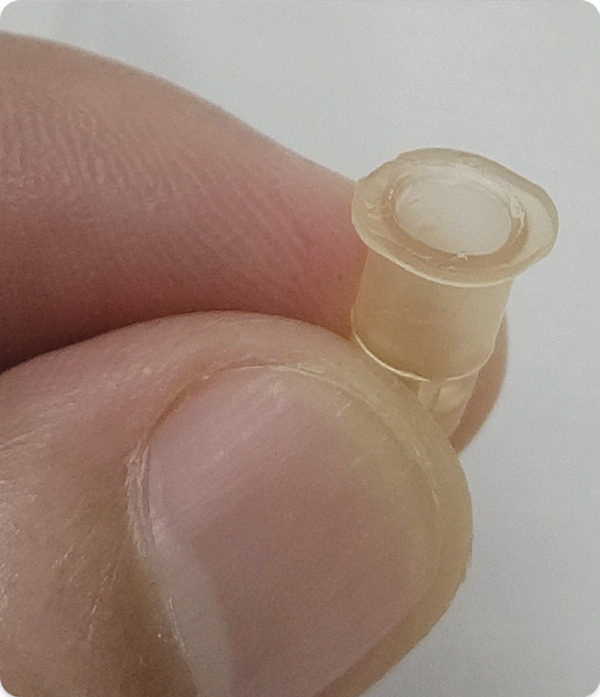
(b)

**Figure:**
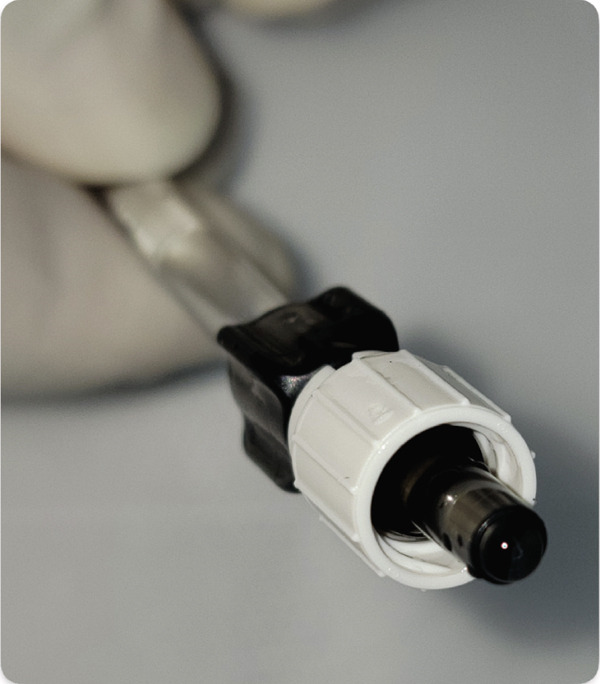
(c)

The B‐BANG technique was performed just like the traditional BANG procedure by one of the authors (M.I.I.), but it was modified by connecting an irrigating cannula from a phacoemulsification machine to a 26‐G needle bent at the tip (Figure 2) [9]. The AC was filled with 4% hydroxypropyl methyl cellulose (HPMC) after creating a 2.4‐mm temporal clear corneal incision. Both the patient’s head and the microscope’s heads were angled at 45° and 35°, respectively. A surgical gonio lens was used to visualize the nasal TM. A 26‐G bent needle tip was inserted into the eye, and goniotomy was performed in the nasal, inferior, and potentially favorable quadrants. In the case of a 360° goniotomy (those with ≥ 3 AGM and moderate glaucomatous damage), a 2.4‐mm potentially favorable clear corneal incision was also created to complete the temporal goniotomy. During the procedure, the bottle height was kept at the maximum height (100 cm) possible in the Visco mode of the phaco machine, with continuous irrigation to ensure clear visualization of the TM by flushing out blood during goniotomy, allowing precise excision of the TM segment. Viscoelastic was removed from the eye with the use of a coaxial irrigation/aspiration device. Finally, the incisions were hydrated with a balanced salt solution (BSS) and sealed. The extent of goniotomy was estimated intraoperatively using visual reference to angle clock hours and recorded in degrees.

Figure 2(a–c) Attachment of bent 26‐G needle with irrigation tube of phaco machine.

**Figure:**
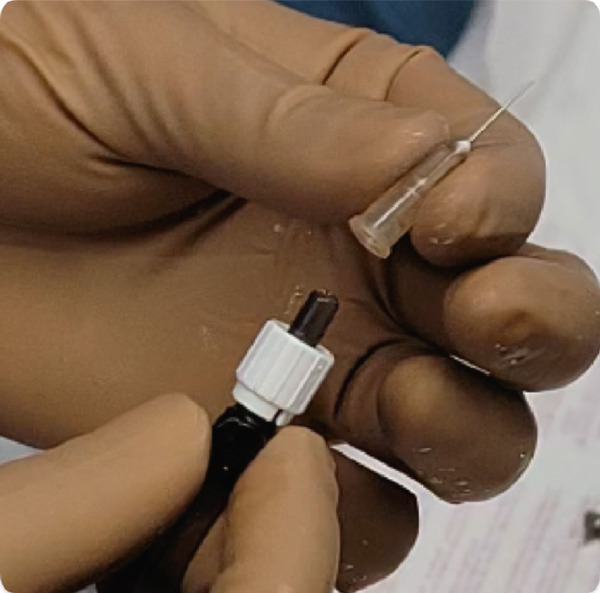
(a)

**Figure:**
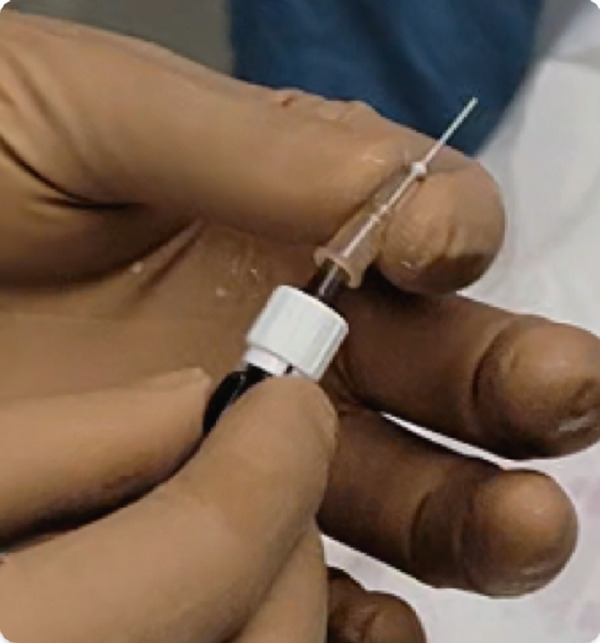
(b)

**Figure:**
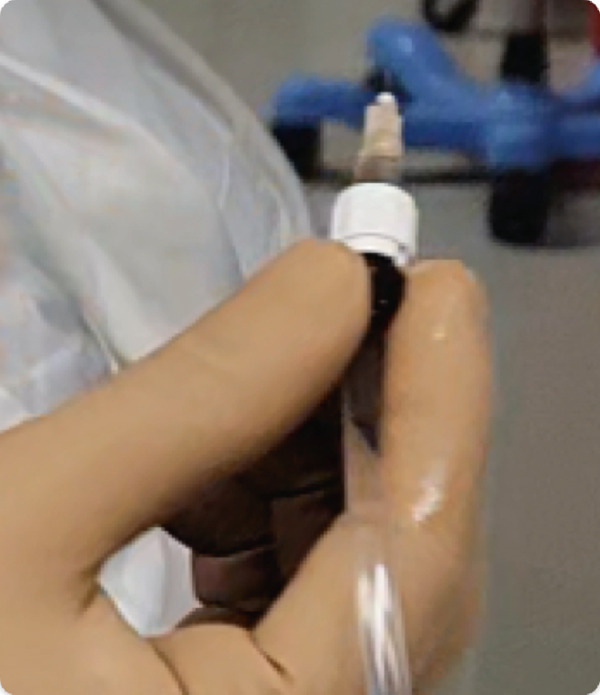
(c)

### 2.4. Postoperative Care

After surgery, all patients received 5% moxifloxacin q.i.d. for a month and 1% prednisolone acetate hourly for a day. Prednisolone was then tapered as follows: every 2 h for 7 days, every 4 h for 7 days, every 6 h for 7 days, and every 8 h for 7 days. Additionally, 2% pilocarpine was continued twice daily for 2 weeks. If the IOP remained ≥ 20 mmHg postoperatively, AGMs were to be added; however, none of the patients required them during the follow‐up period.

### 2.5. Follow‐Up

Following surgery, patients were monitored for 1, 3, and 6 months. All follow‐up visits included records of BCVA, IOP, and AGM usage. A topical carbonic anhydrase inhibitor (CAI) was recommended if the IOP crossed 20 mmHg. Patients continued their routine follow‐up under the operating surgeon’s (M.I.I.) care after completing the trial endpoint.

### 2.6. Statistical Analysis

Stata Version 17.0 (StataCorp, College Station, Texas, United States) was used for all statistical analyses. Statistical analysis was performed using paired *t*‐tests and Wilcoxon signed‐rank tests as a nonparametric sensitivity analysis. Normality was assessed using the Shapiro–Wilk test. Effect sizes were reported using Cohen’s *d* with 95% confidence intervals (CIs). Changes in AGM counts were analyzed descriptively. A descriptive subgroup analysis was also conducted between standalone B‐BANG and combined phacoemulsification with B‐BANG. A *p* value of < 0.05 was considered statistically significant.

### 2.7. Outcome Measures

Outcomes were reported as mean IOP at 1, 3, and 6 months and mean change in the number of IOP‐lowering medications at follow‐up. To report safety, intraoperative and postoperative complications were also noted. Surgical success was defined according to modified World Glaucoma Association criteria: (a) complete success: postoperative IOP < 21 mmHg and ≥ 20% reduction from baseline without any AGMs at 6 months, (b) qualified success: postoperative IOP < 21 mmHg and ≥ 20% reduction from baseline with or without AGMs at 6 months, and (c) failure: IOP ≥ 21 mmHg or < 20% reduction from baseline or need for further glaucoma surgery [10].

## 3. Results

### 3.1. Patient Demographics and Baseline Characteristics

A total of 11 patients were included in the study, with a mean age of 44.0 ± 12.4 years (range: 29–70 years). The majority of patients were female (54.55%, *n* = 6), and the rest were male (45.45%, *n* = 5). Regarding the eye involved, 63.64% (*n* = 7) of cases affected the right eye, while 36.36% (*n* = 4) involved the left eye. Concerning glaucoma severity, 54.55% of patients had mild POAG, and 45.45% had moderate POAG. Other parameters are shown in Table 1.

**Table 1:** Patient demographics and baseline characteristics.

**Variable**	**M** **e** **a** **n** ± **S** **D**	**Count (%)**
Age (years)	44.0 ± 12.4	
Sex		
Male		5 (45.5%)
Female		6 (54.5%)
POAG severity		
Mild		6 (54.5%)
Moderate		5 (45.5%)
Preoperative IOP (mmHg)	22.73 ± 7.38	
C‐D ratio	0.63 ± 0.09	
AGM	2.09 ± 0.94	
VFI (%)	94.36 ± 3.04	
MD (dB)	−3.73 ± 1.94	
PSD (dB)	3.74 ± 1.82	
Average RNFL thickness (*μ*m)	88.82 ± 8.3	

Abbreviations: AGM, antiglaucoma medication; C‐D, cup‐to‐disc; IOP, intraocular pressure; MD, mean deviation; POAG, primary open‐angle glaucoma; PSD, pattern standard deviation; RNFL, retinal nerve fiber layer; SD, standard deviation; VFI, visual field index.

### 3.2. Postoperative IOP Outcomes

The mean preoperative IOP was 22.73 ± 7.38 mmHg. Postoperatively, mean IOP significantly decreased to 16.45 ± 3.27 mmHg at 1 month (*Δ*: −6.27 mmHg, 95% CI: −10.25 to −2.30, *p* = 0.0056, Cohen’s *d* = 1.06), 15.27 ± 2.20 mmHg at 3 months (*Δ*: −7.45 mmHg, 95% CI: −12.92 to −1.99, *p* = 0.0125, *d* = 0.92), and 17.00 ± 2.19 mmHg at 6 months (*Δ*: −5.73 mmHg, 95% CI: −11.03 to −0.43, *p* = 0.0368, *d* = 0.73).

**Figure 3:**
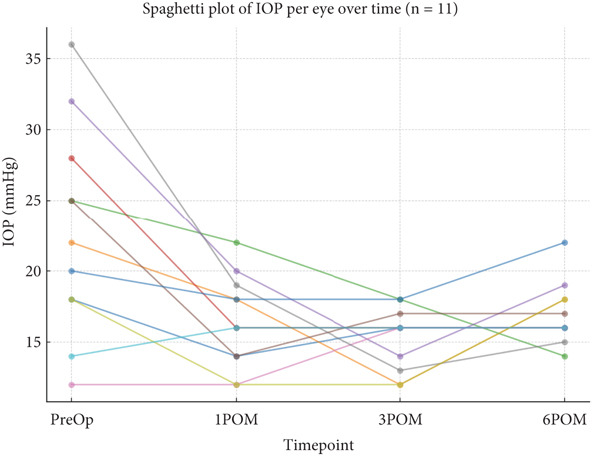
Individual IOP trajectories before and after B‐BANG (IOP, intraocular pressure; POM, postoperative month).

Effect sizes ranged from medium to large, supporting meaningful clinical impact. A spaghetti plot (Figure 3) illustrates per‐eye IOP trajectories across time points, highlighting consistent reduction with minor interindividual variation.

### 3.3. AGM Usage

The mean number of AGMs preoperatively was 2.09 ± 0.94. This significantly dropped to 0.27 ± 0.47 at 1M and 3M (*p* = 0.0078, *d* = 2.42) and 0.45 ± 0.52 at 6M (*p* = 0.0313, *d* = 2.02).

**Figure 4:**
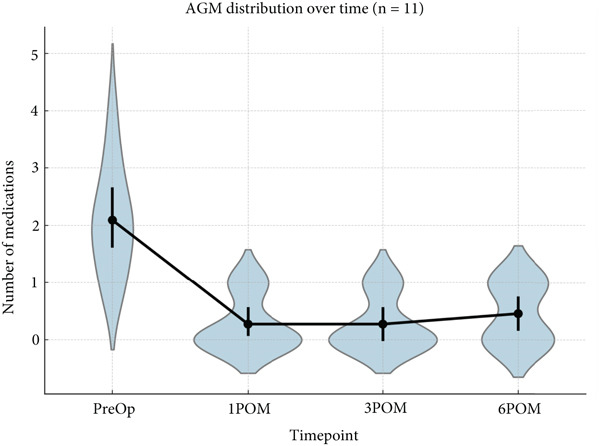
Distribution of AGM usage at each follow‐up visit (AGM, antiglaucoma medication; POM, postoperative month).

A violin and point plot (Figure 4) shows how medication burden sharply reduced postoperatively, with converging distribution across eyes.

### 3.4. Medication Endpoint Summary

At 6 months, 6 of 11 eyes (54.5%) were completely medication‐free (95% CI: 23.4%–83.3%), and all 11 eyes (100%) achieved at least a 1‐medication reduction (95% CI: 71.5%–100.0%).

### 3.5. Subgroup Analysis: IOP and AGM

A subgroup analysis was performed comparing outcomes between eyes undergoing standalone B‐BANG (*n* = 6, mild POAG) and combined phaco + B‐BANG (*n* = 5, moderate POAG) (Table 2).

**Table 2:** Subgroup comparison of IOP and AGM outcomes between standalone B‐BANG and combined phaco + B‐BANG procedures (IOP, intraocular pressure; AGM, antiglaucoma medication; POM, postoperative month).

**Outcome**	**Time point**	**Standalone B-BANG (** **n** = 6**)**	**Combined phaco + B-BANG (** **n** = 5**)**	**p** **value (Wilcoxon)**	**Cohen’s** **d**
IOP (mmHg)	Preop	22.50 ± 8.43	23.00 ± 6.82	0.9168	−0.06
	1POM	15.67 ± 3.39	17.40 ± 3.05	0.3694	−0.54
	3POM	14.50 ± 2.74	16.20 ± 1.92	0.2906	−0.72
	6POM	16.33 ± 1.86	17.80 ± 2.17	0.3694	−0.72

AGM	Preop	2.00 ± 0.63	2.20 ± 1.10	0.5981	−0.22
	1POM–6POM	0.33 ± 0.52	0.60 ± 0.55	0.3694	−0.50

Preoperative IOP was comparable between groups (22.50 ± 8.43 vs. 23.00 ± 6.82 mmHg; *p* = 0.9168). At 6 months, mean IOP was slightly lower in the standalone group (16.33 ± 1.86 vs. 17.80 ± 2.17 mmHg; *p* = 0.3694; Cohen’s *d* = –0.72). Similarly, AGM use at 6 months favored the standalone group (0.33 ± 0.52 vs. 0.60 ± 0.55; *p* = 0.3694).

At all postoperative time points, the standalone B‐BANG group demonstrated numerically lower IOP and AGM usage compared to the combined group, though these differences did not reach statistical significance.

While the trends suggest potential benefit with standalone B‐BANG, the small sample size limits firm statistical conclusions. Effect sizes (Cohen’s *d*) ranged from small to moderate.

### 3.6. Safety Outcomes

Only three cases (27%) experienced mild intraoperative hyphema, resolving spontaneously. No eyes experienced IOP spikes ≥ 10 mmHg, AC inflammation, or required reoperation.

## 4. Discussion

Recently, the range of surgical procedures available for glaucoma has significantly expanded. They exhibit increased selectivity and precision regarding the pathological factors associated with the progression of glaucoma. MIGS has become increasingly favored in the field of glaucoma management globally, as it offers a safer alternative to traditional filtration surgeries while demonstrating greater efficacy than pharmacological or laser interventions [5]. Many of these can be combined with cataract surgery without increasing the risk of standard phacoemulsification [10]. Nevertheless, the expense associated with MIGS devices renders these procedures unattainable for a significant portion of patients and surgeons globally, including those in Bangladesh. Conversely, the BANG presents a cost‐efficient option compared to numerous TM‐based glaucoma interventions, which employ a conventional hypodermic needle modified to facilitate the excision of the TM. However, there is an issue with hyphema during the procedure, which precludes the view of the angle and thus requires frequent OVDs to ensure proper visualization [9].

The results of this study suggest that the B‐BANG technique utilizing a 26‐G hypodermic needle is an effective and cost‐efficient alternative to the more expensive TrabEx+ device for ab interno goniotomy in patients with open‐angle glaucoma. The continuous irrigation provided by the phaco machine’s irrigating cannula in the B‐BANG technique not only enhanced intraoperative visualization by clearing blood from the operative field but also contributed to significant reductions in both IOP and AGM usage at 1, 3, and 6 months postoperatively. A literature‐based comparison of B‐BANG with BANG and TrabEx+ is provided in Table 3 [2, 9].

**Table 3:** Cross‐study comparison of outcomes following B‐BANG, BANG, and TrabEx+.

**Parameter**	**B-BANG (6POM)**	**BANG (6POM)**	**TrabEx+ (12–24POM)**	**Notes**
Preoperative IOP (mmHg)	22.73 ± 7.38	23.00 ± 6.82	31.3 ± 7.3	—
Postoperative IOP (mmHg)	17.00 ± 2.19	13.3 ± 2.5	20.9 ± 10.4	—
IOP reduction (%)	25.2%	73*%* ≥ 20*%* reduction	34% reduction	—
Mean medication use–preop	2.09 ± 0.94	0.5 ± 0.8	2.9 ± 1.2	—
Mean medication use–postop	0.45 ± 0.52	0.5 ± 0.8	1.9 ± 1.3	—
≥1 med reduction (%)	100%	73%	NR	Binomial CI: B‐BANG = 71.5%–100%
Medication‐free (%)	54.5%	73%	NR	B‐BANG = 23.4%–83.3%
Complications	Mild hyphema (27%)	NR	Hyphema 17%, uveitis 3%, hypotony 1%, reoperation 18%	—

### 4.1. Efficacy in IOP Reduction

The mean IOP reduction with the B‐BANG technique was 25.2% at 6 months, which is comparable to the 33.2% reduction reported for TrabEx+ at 24 months in a retrospective case series but had a higher preoperative IOP baseline [2]. Similar procedures using KDB combined with phacoemulsification have been shown to reduce IOP by up to 26.2% over 12 months [11]. In contrast, the original BANG technique demonstrated a 23% reduction in IOP at 6 months, suggesting that the enhanced visualization provided by the continuous irrigation system in B‐BANG may contribute to more precise and effective TM excision without compromising surgical efficacy [9].

In the case of B‐BANG, statistically significant reductions were observed at all time points: 1 month (*p* = 0.0056, Cohen’s *d* = 1.06), 3 months (*p* = 0.0125, *d* = 0.92), and 6 months (*p* = 0.0368, *d* = 0.73), with effect sizes supporting meaningful clinical improvements. The inclusion of 95% CIs further supports the consistency and reliability of IOP reduction. Although IOP values slightly rebounded by 6 months, most probably due to the development of segmental PAS, higher pigmentation of TM, and preoperative higher IOP, the reduction remained clinically relevant [12–15]. A per‐eye spaghetti plot (Figure 3) showed stable trends across patients.

### 4.2. AGM Reduction and Medication Independence

The reduction in AGM usage was substantial in B‐BANG, with mean use decreasing from 2.09 ± 0.94 to 0.45 ± 0.52, which is consistent with results reported for TrabEx+, 2.9 ± 1.2 to 1.9 ± 1.3 AGMs at the latest follow‐up (*p* < 0.001) [2]. A meta‐analysis by Kaplowitz et al. also showed a medication reduction to 1.0 from the baseline medications in 14 studies at the 2‐year follow‐up [16]. In comparison, the original BANG technique also demonstrated a reduction in AGM dependence but was limited by transient obscuration of the operative field due to bleeding [9].

At 6 months, 54.5% of patients were medication‐free, and all (100%) achieved at least one medication reduction (95% CI: 71.5%–100%) in the case of B‐BANG. The violin plot (Figure 4) highlighted both the extent and convergence of medication reduction across patients, reinforcing the consistency of the effect.

Compared to TrabEx+ and BANG, B‐BANG performed comparably or better in reducing medication burden, with fewer patients requiring medications postoperatively. B‐BANG covered a wider extent of goniotomy (253.64°) compared to BANG (102.4°) and TrabEx+ (90°–180°); this variation may explain the greater reduction in medication usage observed in B‐BANG [2, 9].

### 4.3. Subgroup Trends: IOP and AGM

Subgroup analysis showed numerically lower IOP and AGM usage in the standalone B‐BANG group compared to the combined phaco and B‐BANG, though these differences were not statistically significant. This trend may suggest that standalone procedures are equally or more effective for mild cases, although further study is required. As this pilot study was not powered for subgroup comparisons, these results should be interpreted cautiously.

### 4.4. Visualization and Intraoperative Efficiency

The primary advantage of B‐BANG over BANG lies in its potentially favorable intraoperative visualization. The continuous irrigation system effectively clears blood from the AC angle, a limitation noted in BANG, which relies on viscoelastic for temporary blood removal and visualization [2]. Enhanced visualization likely contributes to the more precise removal of the TM, thereby improving surgical outcomes and reducing the risk of residual debris obstructing outflow channels.

TrabEx+ utilizes an integrated irrigation and aspiration system designed to maintain a clear field during goniectomy by simultaneously removing blood and debris [2, 17]. However, the high cost of this device limits its accessibility in resource‐limited settings [2, 8, 17]. In contrast, B‐BANG utilizes standard phaco equipment, making it a more practical option for low‐cost settings without compromising visualization quality.

### 4.5. Safety Profile and Complications

The B‐BANG technique demonstrated a favorable safety profile. In comparison, TrabEx+ was associated with a 17% rate of hyphema and minor complications such as uveitis (3%) and hypotony (1%), highlighting a key advantage of B‐BANG in minimizing postoperative risks [2].

The original BANG technique, while also demonstrating a low complication rate, was limited by temporary obscuration of the operative field due to bleeding, a challenge effectively addressed by the continuous irrigation in B‐BANG by providing uninterrupted visibility of the TM and collector channels, potentially reducing the risk of incomplete excision and minimizing the risk of hyphema, a common complication noted with other ab interno techniques [9, 18].

Only mild, self‐limiting intraoperative hyphema was observed in three cases (27%), and no postoperative complications such as IOP spikes ≥ 10 mmHg, inflammation, or need for reoperation were reported over 6 months in B‐BANG cases. These outcomes suggest that B‐BANG is a low‐risk intervention when performed with appropriate irrigation and visualization techniques.

### 4.6. Lower Material Cost and Accessibility

One of the most significant advantages of B‐BANG is its lower cost by using a 26‐G hypodermic needle. The use of standard phaco equipment and an irrigating cannula significantly reduces procedural costs compared to the high cost of the TrabEx+ device. The cost‐prohibitive nature of TrabEx+ has been cited as a barrier to its widespread adoption, especially in low‐ and middle‐income countries [2].

The affordability of B‐BANG, combined with its effective IOP and AGM reduction capabilities, positions it as an ideal choice for glaucoma management in resource‐limited settings. This cost advantage, coupled with its simplified setup and effective visualization, makes B‐BANG a practical alternative to TrabEx+ and other MIGS techniques, especially for surgeons in less developed healthcare systems. It can be incorporated with the BSS line hanging on a stand where a phaco machine is not available but make sure to maintain the highest bottle height possible while maintaining a sustained pressure to release BSS within the AC continuously.

## 5. Conclusions

B‐BANG, a low‐cost modification of BANG, demonstrated favorable midterm outcomes in this pilot study, with reductions in IOP and AGM use over 6 months. The procedure was feasible in both standalone and combined phacoemulsification settings, with a low rate of intraoperative complications. These findings should be interpreted with caution given the study’s limitations, including small sample size, single‐surgeon experience, short follow‐up, and absence of masked outcome assessment. The influence of cataract surgery in the combined group and the modest rise in IOP at 6 months require further investigation. Future research should incorporate prospective registries, randomized comparisons with BANG and TrabEx+, and structured economic evaluations to better assess the clinical utility and cost‐effectiveness of B‐BANG.

## Ethics Statement

The Bangladesh Eye Hospital’s Institutional Review Board approved the study, IRB No. BEH/2023/07/2.

## Consent

All participants in the study provided informed consent.

## Conflicts of Interest

The authors declare no conflicts of interest.

## Author Contributions

M.I.I.: conceptualization, methodology, validation, investigation, data curation, and writing—original draft preparation, review, and editing. F.O.: formal analysis, data curation, editing, and review.

## Funding

No funding was received for this manuscript.

## Data Availability

The corresponding author can provide data supporting this study’s findings upon request.
